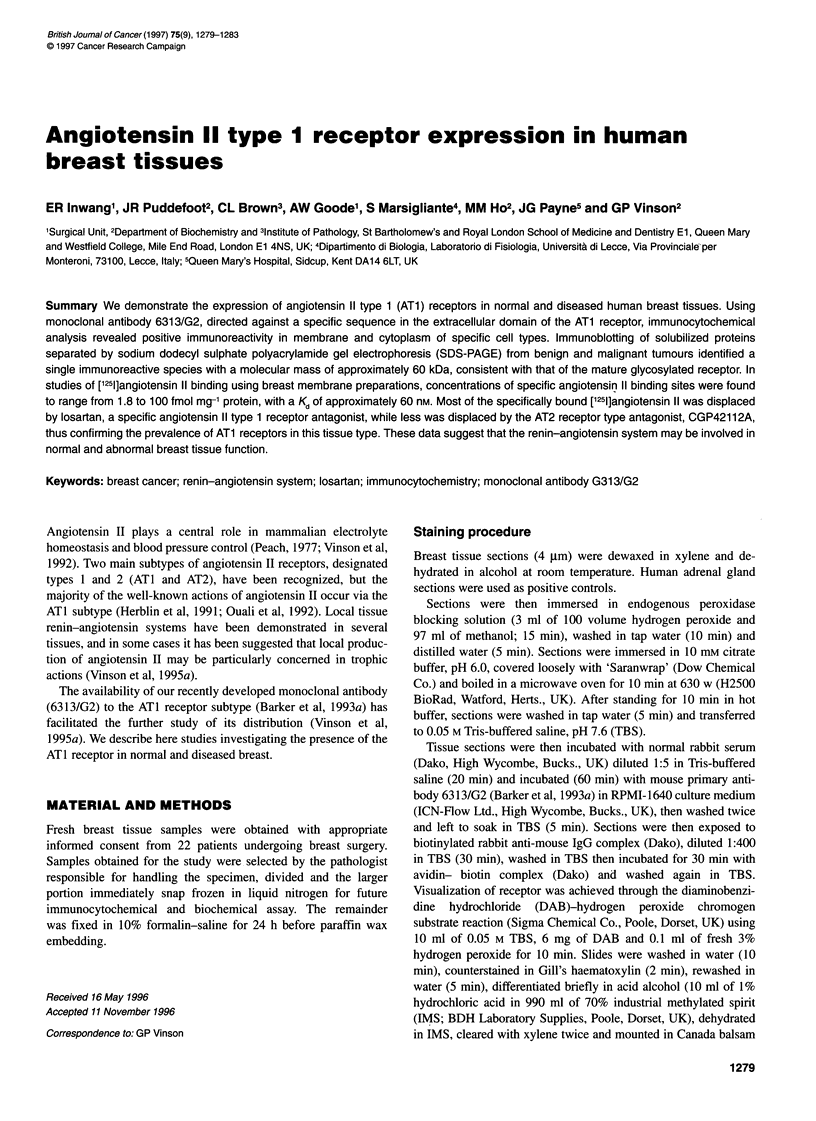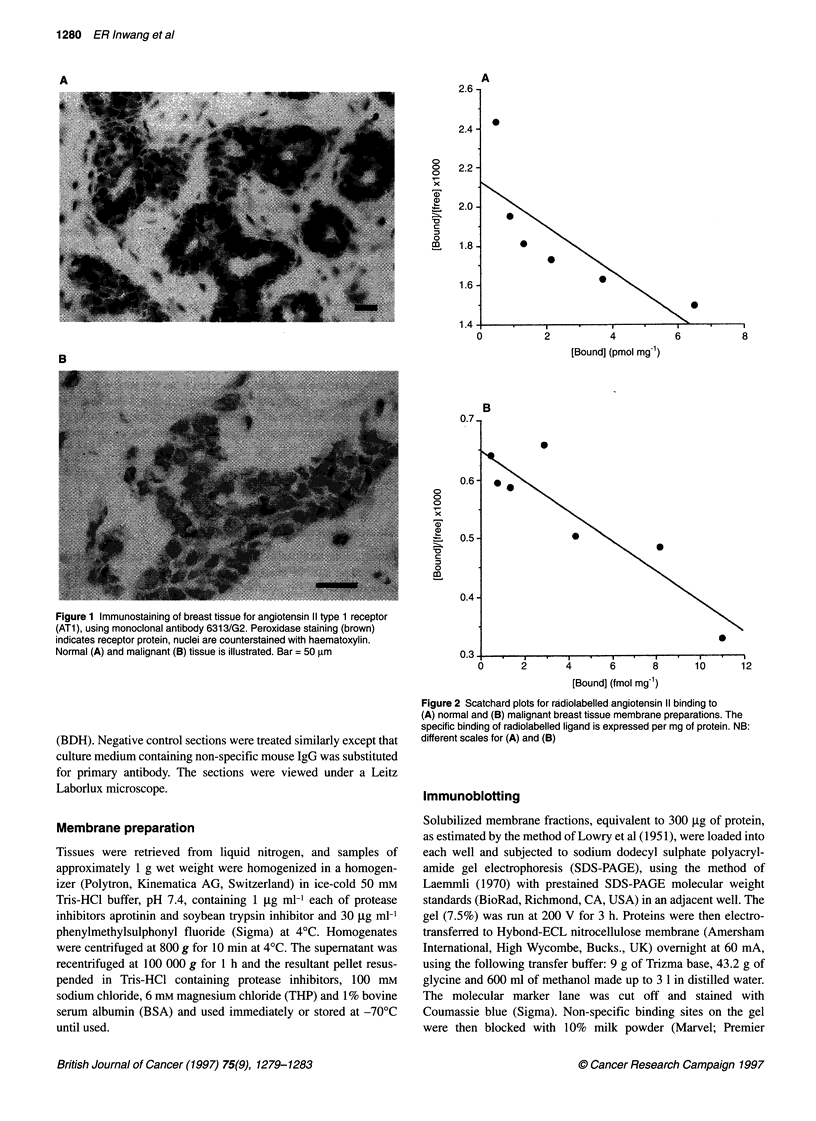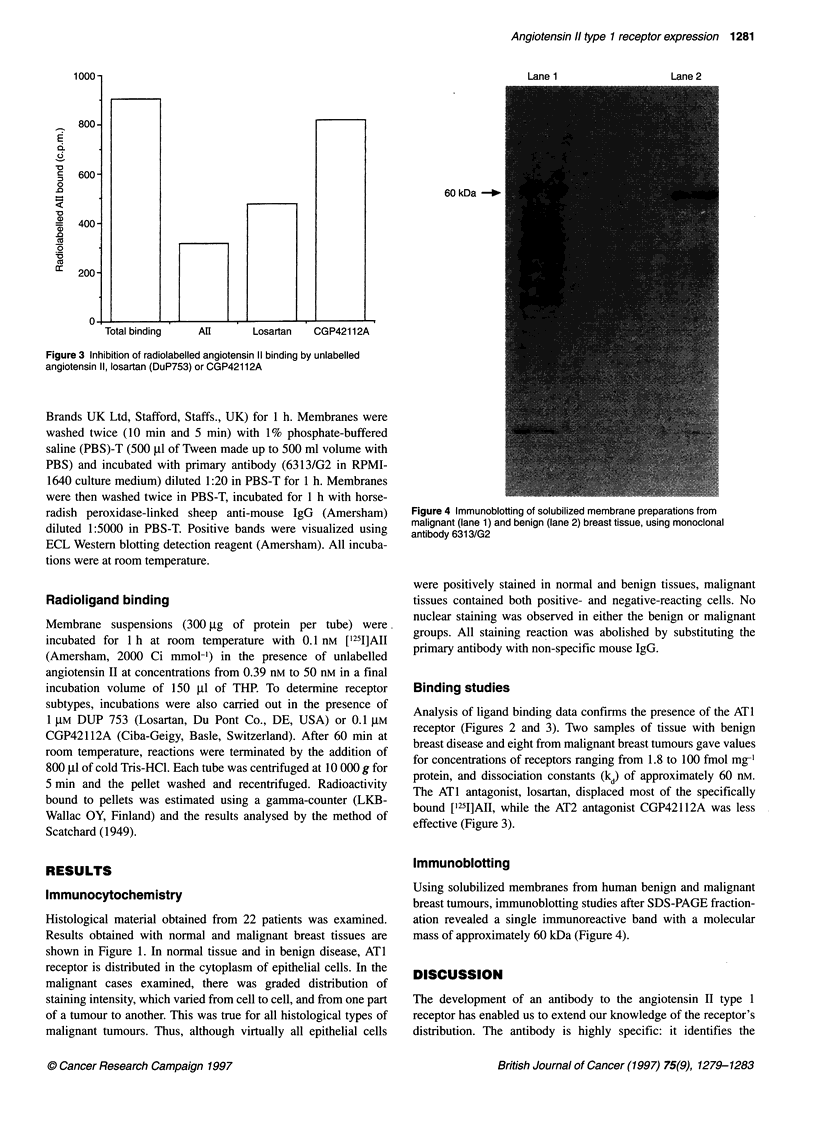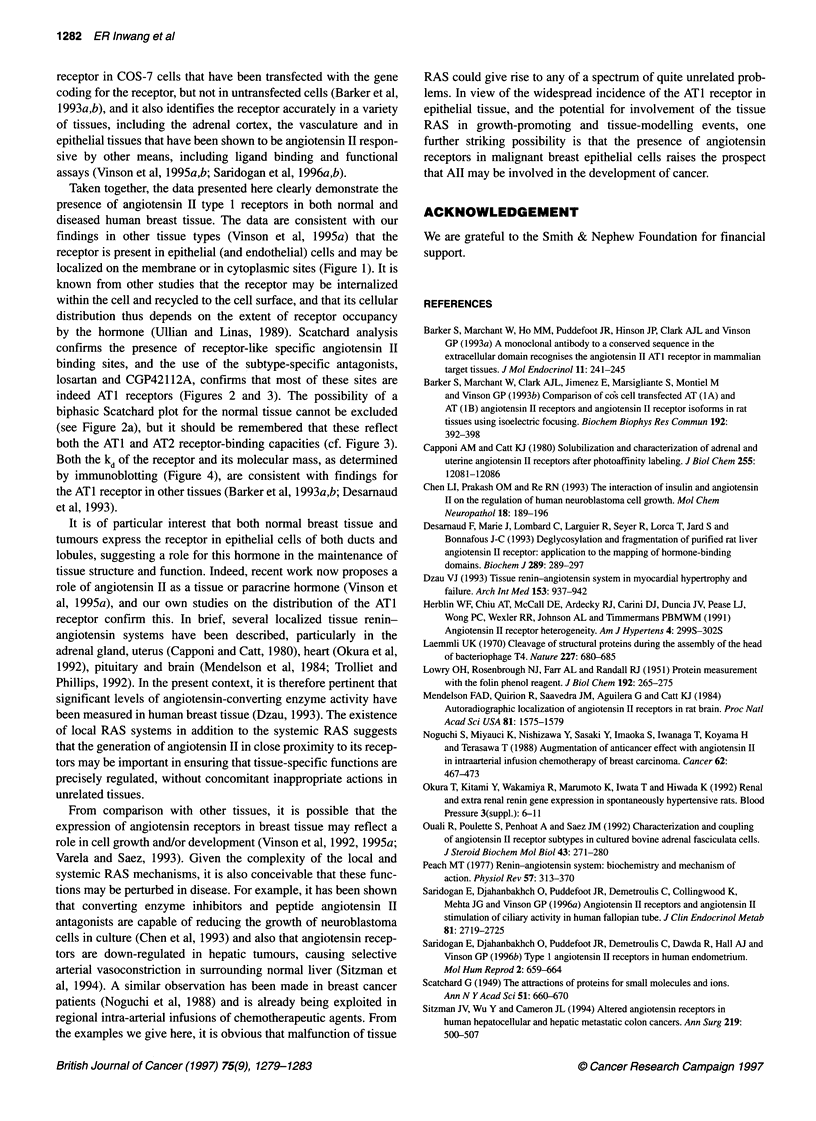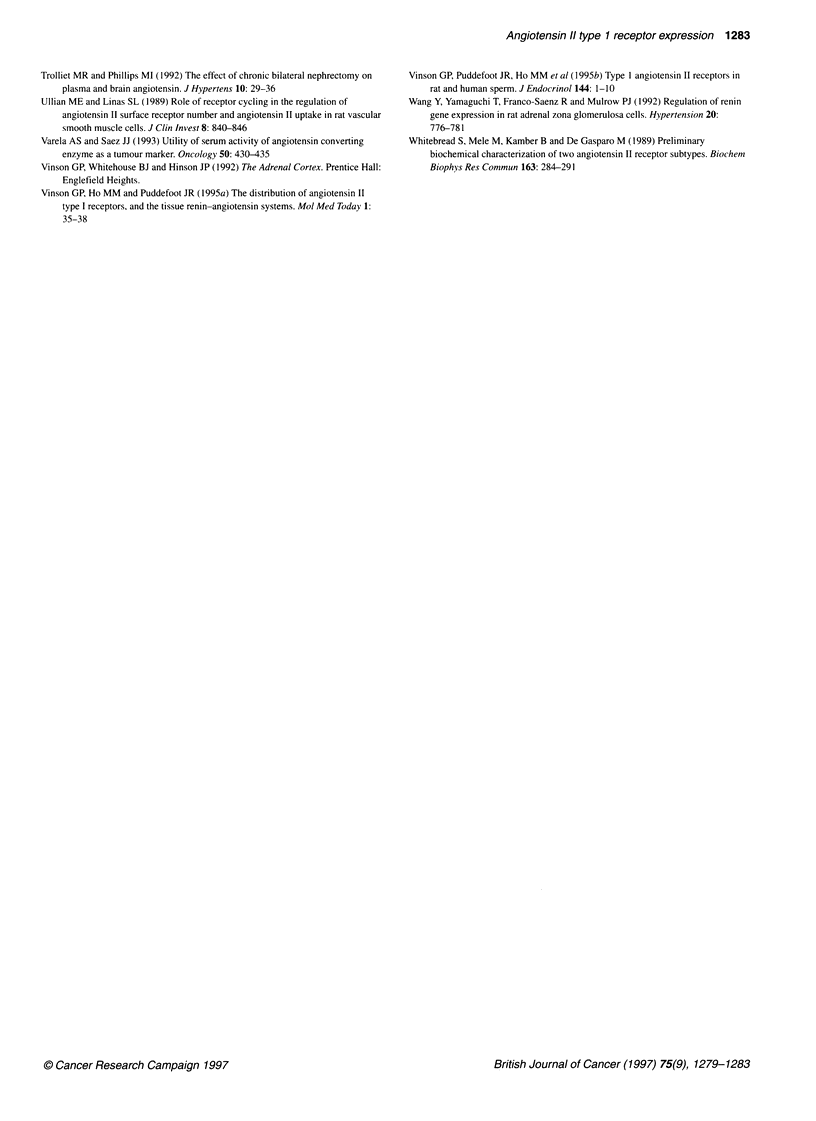# Angiotensin II type 1 receptor expression in human breast tissues.

**DOI:** 10.1038/bjc.1997.217

**Published:** 1997

**Authors:** E. R. Inwang, J. R. Puddefoot, C. L. Brown, A. W. Goode, S. Marsigliante, M. M. Ho, J. G. Payne, G. P. Vinson

**Affiliations:** Surgical Unit, St Bartholomew's and Royal London School of Medicine and Dentistry E1, Queen Mary and Westfield College, UK.

## Abstract

**Images:**


British Journal of Cancer (1997) 75(9), 1279–1283
© 1997 Cancer Research Campaign

### Angiotensin II type 1 receptor expression in human breast tissues

**ER Inwang[1], JR Puddefoot[2], CL Brown[3], AW Goode[1], S Marsigliante[4], MM Ho[2], JG Payne[5] and GP Vinson[2]**

[1]Surgical Unit, [2]Department of Biochemistry and [3]Institute of Pathology, St Bartholomew's and Royal London School of Medicine and Dentistry E1, Queen Mary and Westfield College, Mile End Road, London E1 4NS, UK; [4]Dipartimento di Biologia, Laboratorio di Fisiologia, Università di Lecce, Via Provinciale per Monteroni, 73100, Lecce, Italy; [5]Queen Mary's Hospital, Sidcup, Kent DA14 6LT, UK

**Summary** We demonstrate the expression of angiotensin II type 1 (AT1) receptors in normal and diseased human breast tissues. Using monoclonal antibody 6313/G2, directed against a specific sequence in the extracellular domain of the AT1 receptor, immunocytochemical analysis revealed positive immunoreactivity in membrane and cytoplasm of specific cell types. Immunoblotting of solubilized proteins separated by sodium dodecyl sulphate polyacrylamide gel electrophoresis (SDS-PAGE) from benign and malignant tumours identified a single immunoreactive species with a molecular mass of approximately 60 kDa, consistent with that of the mature glycosylated receptor. In studies of [$^{125}$I]angiotensin II binding using breast membrane preparations, concentrations of specific angiotensin II binding sites were found to range from 1.8 to 100 fmol mg$^{-1}$ protein, with a $K_d$ of approximately 60 nM. Most of the specifically bound [$^{125}$I]angiotensin II was displaced by losartan, a specific angiotensin II type 1 receptor antagonist, while less was displaced by the AT2 receptor type antagonist, CGP42112A, thus confirming the prevalence of AT1 receptors in this tissue type. These data suggest that the renin–angiotensin system may be involved in normal and abnormal breast tissue function.

**Keywords:** breast cancer; renin–angiotensin system; losartan; immunocytochemistry; monoclonal antibody G313/G2

Angiotensin II plays a central role in mammalian electrolyte homeostasis and blood pressure control (Peach, 1977; Vinson et al, 1992). Two main subtypes of angiotensin II receptors, designated types 1 and 2 (AT1 and AT2), have been recognized, but the majority of the well-known actions of angiotensin II occur via the AT1 subtype (Herblin et al, 1991; Ouali et al, 1992). Local tissue renin–angiotensin systems have been demonstrated in several tissues, and in some cases it has been suggested that local production of angiotensin II may be particularly concerned in trophic actions (Vinson et al, 1995*a*).

The availability of our recently developed monoclonal antibody (6313/G2) to the AT1 receptor subtype (Barker et al, 1993*a*) has facilitated the further study of its distribution (Vinson et al, 1995*a*). We describe here studies investigating the presence of the AT1 receptor in normal and diseased breast.

### MATERIAL AND METHODS

Fresh breast tissue samples were obtained with appropriate informed consent from 22 patients undergoing breast surgery. Samples obtained for the study were selected by the pathologist responsible for handling the specimen, divided and the larger portion immediately snap frozen in liquid nitrogen for future immunocytochemical and biochemical assay. The remainder was fixed in 10% formalin–saline for 24 h before paraffin wax embedding.

*Received 16 May 1996*
*Accepted 11 November 1996*

*Correspondence to:* GP Vinson

### Staining procedure

Breast tissue sections (4 μm) were dewaxed in xylene and dehydrated in alcohol at room temperature. Human adrenal gland sections were used as positive controls.

Sections were then immersed in endogenous peroxidase blocking solution (3 ml of 100 volume hydrogen peroxide and 97 ml of methanol; 15 min), washed in tap water (10 min) and distilled water (5 min). Sections were immersed in 10 mM citrate buffer, pH 6.0, covered loosely with 'Saranwrap' (Dow Chemical Co.) and boiled in a microwave oven for 10 min at 630 w (H2500 BioRad, Watford, Herts., UK). After standing for 10 min in hot buffer, sections were washed in tap water (5 min) and transferred to 0.05 M Tris-buffered saline, pH 7.6 (TBS).

Tissue sections were then incubated with normal rabbit serum (Dako, High Wycombe, Bucks., UK) diluted 1:5 in Tris-buffered saline (20 min) and incubated (60 min) with mouse primary antibody 6313/G2 (Barker et al, 1993*a*) in RPMI-1640 culture medium (ICN-Flow Ltd., High Wycombe, Bucks., UK), then washed twice and left to soak in TBS (5 min). Sections were then exposed to biotinylated rabbit anti-mouse IgG complex (Dako), diluted 1:400 in TBS (30 min), washed in TBS then incubated for 30 min with avidin– biotin complex (Dako) and washed again in TBS. Visualization of receptor was achieved through the diaminobenzidine hydrochloride (DAB)–hydrogen peroxide chromogen substrate reaction (Sigma Chemical Co., Poole, Dorset, UK) using 10 ml of 0.05 M TBS, 6 mg of DAB and 0.1 ml of fresh 3% hydrogen peroxide for 10 min. Slides were washed in water (10 min), counterstained in Gill's haematoxylin (2 min), rewashed in water (5 min), differentiated briefly in acid alcohol (10 ml of 1% hydrochloric acid in 990 ml of 70% industrial methylated spirit (IMS; BDH Laboratory Supplies, Poole, Dorset, UK), dehydrated in IMS, cleared with xylene twice and mounted in Canada balsam

**Figure 1** Immunostaining of breast tissue for angiotensin II type 1 receptor (AT1), using monoclonal antibody 6313/G2. Peroxidase staining (brown) indicates receptor protein, nuclei are counterstained with haematoxylin. Normal (**A**) and malignant (**B**) tissue is illustrated. Bar = 50 μm

**Figure 2** Scatchard plots for radiolabelled angiotensin II binding to (**A**) normal and (**B**) malignant breast tissue membrane preparations. The specific binding of radiolabelled ligand is expressed per mg of protein. NB: different scales for (**A**) and (**B**)

(BDH). Negative control sections were treated similarly except that culture medium containing non-specific mouse IgG was substituted for primary antibody. The sections were viewed under a Leitz Laborlux microscope.

### Membrane preparation

Tissues were retrieved from liquid nitrogen, and samples of approximately 1 g wet weight were homogenized in a homogenizer (Polytron, Kinematica AG, Switzerland) in ice-cold 50 mM Tris-HCl buffer, pH 7.4, containing 1 μg $ml^{-1}$ each of protease inhibitors aprotinin and soybean trypsin inhibitor and 30 μg $ml^{-1}$ phenylmethylsulphonyl fluoride (Sigma) at 4°C. Homogenates were centrifuged at 800 ***g*** for 10 min at 4°C. The supernatant was recentrifuged at 100 000 ***g*** for 1 h and the resultant pellet resuspended in Tris-HCl containing protease inhibitors, 100 mM sodium chloride, 6 mM magnesium chloride (THP) and 1% bovine serum albumin (BSA) and used immediately or stored at –70°C until used.

### Immunoblotting

Solubilized membrane fractions, equivalent to 300 μg of protein, as estimated by the method of Lowry et al (1951), were loaded into each well and subjected to sodium dodecyl sulphate polyacrylamide gel electrophoresis (SDS-PAGE), using the method of Laemmli (1970) with prestained SDS-PAGE molecular weight standards (BioRad, Richmond, CA, USA) in an adjacent well. The gel (7.5%) was run at 200 V for 3 h. Proteins were then electrotransferred to Hybond-ECL nitrocellulose membrane (Amersham International, High Wycombe, Bucks., UK) overnight at 60 mA, using the following transfer buffer: 9 g of Trizma base, 43.2 g of glycine and 600 ml of methanol made up to 3 l in distilled water. The molecular marker lane was cut off and stained with Coumassie blue (Sigma). Non-specific binding sites on the gel were then blocked with 10% milk powder (Marvel; Premier

© Cancer Research Campaign 1997

**Figure 3** Inhibition of radiolabelled angiotensin II binding by unlabelled angiotensin II, losartan (DuP753) or CGP42112A

Brands UK Ltd, Stafford, Staffs., UK) for 1 h. Membranes were washed twice (10 min and 5 min) with 1% phosphate-buffered saline (PBS)-T (500 µl of Tween made up to 500 ml volume with PBS) and incubated with primary antibody (6313/G2 in RPMI-1640 culture medium) diluted 1:20 in PBS-T for 1 h. Membranes were then washed twice in PBS-T, incubated for 1 h with horse-radish peroxidase-linked sheep anti-mouse IgG (Amersham) diluted 1:5000 in PBS-T. Positive bands were visualized using ECL Western blotting detection reagent (Amersham). All incubations were at room temperature.

### Radioligand binding

Membrane suspensions (300 µg of protein per tube) were incubated for 1 h at room temperature with 0.1 nM [$^{125}$I]AII (Amersham, 2000 Ci mmol$^{-1}$) in the presence of unlabelled angiotensin II at concentrations from 0.39 nM to 50 nM in a final incubation volume of 150 µl of THP. To determine receptor subtypes, incubations were also carried out in the presence of 1 µM DUP 753 (Losartan, Du Pont Co., DE, USA) or 0.1 µM CGP42112A (Ciba-Geigy, Basle, Switzerland). After 60 min at room temperature, reactions were terminated by the addition of 800 µl of cold Tris-HCl. Each tube was centrifuged at 10 000 ***g*** for 5 min and the pellet washed and recentrifuged. Radioactivity bound to pellets was estimated using a gamma-counter (LKB-Wallac OY, Finland) and the results analysed by the method of Scatchard (1949).

### RESULTS

### Immunocytochemistry

Histological material obtained from 22 patients was examined. Results obtained with normal and malignant breast tissues are shown in Figure 1. In normal tissue and in benign disease, AT1 receptor is distributed in the cytoplasm of epithelial cells. In the malignant cases examined, there was graded distribution of staining intensity, which varied from cell to cell, and from one part of a tumour to another. This was true for all histological types of malignant tumours. Thus, although virtually all epithelial cells were positively stained in normal and benign tissues, malignant tissues contained both positive- and negative-reacting cells. No nuclear staining was observed in either the benign or malignant groups. All staining reaction was abolished by substituting the primary antibody with non-specific mouse IgG.

**Figure 4** Immunoblotting of solubilized membrane preparations from malignant (lane 1) and benign (lane 2) breast tissue, using monoclonal antibody 6313/G2

### Binding studies

Analysis of ligand binding data confirms the presence of the AT1 receptor (Figures 2 and 3). Two samples of tissue with benign breast disease and eight from malignant breast tumours gave values for concentrations of receptors ranging from 1.8 to 100 fmol mg$^{-1}$ protein, and dissociation constants ($k_d$) of approximately 60 nM. The AT1 antagonist, losartan, displaced most of the specifically bound [$^{125}$I]AII, while the AT2 antagonist CGP42112A was less effective (Figure 3).

### Immunoblotting

Using solubilized membranes from human benign and malignant breast tumours, immunoblotting studies after SDS-PAGE fractionation revealed a single immunoreactive band with a molecular mass of approximately 60 kDa (Figure 4).

### DISCUSSION

The development of an antibody to the angiotensin II type 1 receptor has enabled us to extend our knowledge of the receptor's distribution. The antibody is highly specific: it identifies the

© Cancer Research Campaign 1997

receptor in COS-7 cells that have been transfected with the gene coding for the receptor, but not in untransfected cells (Barker et al, 1993*a*,*b*), and it also identifies the receptor accurately in a variety of tissues, including the adrenal cortex, the vasculature and in epithelial tissues that have been shown to be angiotensin II responsive by other means, including ligand binding and functional assays (Vinson et al, 1995*a*,*b*; Saridogan et al, 1996*a*,*b*).

Taken together, the data presented here clearly demonstrate the presence of angiotensin II type 1 receptors in both normal and diseased human breast tissue. The data are consistent with our findings in other tissue types (Vinson et al, 1995*a*) that the receptor is present in epithelial (and endothelial) cells and may be localized on the membrane or in cytoplasmic sites (Figure 1). It is known from other studies that the receptor may be internalized within the cell and recycled to the cell surface, and that its cellular distribution thus depends on the extent of receptor occupancy by the hormone (Ullian and Linas, 1989). Scatchard analysis confirms the presence of receptor-like specific angiotensin II binding sites, and the use of the subtype-specific antagonists, losartan and CGP42112A, confirms that most of these sites are indeed AT1 receptors (Figures 2 and 3). The possibility of a biphasic Scatchard plot for the normal tissue cannot be excluded (see Figure 2a), but it should be remembered that these reflect both the AT1 and AT2 receptor-binding capacities (cf. Figure 3). Both the $k_d$ of the receptor and its molecular mass, as determined by immunoblotting (Figure 4), are consistent with findings for the AT1 receptor in other tissues (Barker et al, 1993*a*,*b*; Desarnaud et al, 1993).

It is of particular interest that both normal breast tissue and tumours express the receptor in epithelial cells of both ducts and lobules, suggesting a role for this hormone in the maintenance of tissue structure and function. Indeed, recent work now proposes a role of angiotensin II as a tissue or paracrine hormone (Vinson et al, 1995*a*), and our own studies on the distribution of the AT1 receptor confirm this. In brief, several localized tissue renin–angiotensin systems have been described, particularly in the adrenal gland, uterus (Capponi and Catt, 1980), heart (Okura et al, 1992), pituitary and brain (Mendelson et al, 1984; Trolliet and Phillips, 1992). In the present context, it is therefore pertinent that significant levels of angiotensin-converting enzyme activity have been measured in human breast tissue (Dzau, 1993). The existence of local RAS systems in addition to the systemic RAS suggests that the generation of angiotensin II in close proximity to its receptors may be important in ensuring that tissue-specific functions are precisely regulated, without concomitant inappropriate actions in unrelated tissues.

From comparison with other tissues, it is possible that the expression of angiotensin receptors in breast tissue may reflect a role in cell growth and/or development (Vinson et al, 1992, 1995*a*; Varela and Saez, 1993). Given the complexity of the local and systemic RAS mechanisms, it is also conceivable that these functions may be perturbed in disease. For example, it has been shown that converting enzyme inhibitors and peptide angiotensin II antagonists are capable of reducing the growth of neuroblastoma cells in culture (Chen et al, 1993) and also that angiotensin receptors are down-regulated in hepatic tumours, causing selective arterial vasoconstriction in surrounding normal liver (Sitzman et al, 1994). A similar observation has been made in breast cancer patients (Noguchi et al, 1988) and is already being exploited in regional intra-arterial infusions of chemotherapeutic agents. From the examples we give here, it is obvious that malfunction of tissue RAS could give rise to any of a spectrum of quite unrelated problems. In view of the widespread incidence of the AT1 receptor in epithelial tissue, and the potential for involvement of the tissue RAS in growth-promoting and tissue-modelling events, one further striking possibility is that the presence of angiotensin receptors in malignant breast epithelial cells raises the prospect that AII may be involved in the development of cancer.

### ACKNOWLEDGEMENT

We are grateful to the Smith & Nephew Foundation for financial support.

### REFERENCES

Barker S, Marchant W, Ho MM, Puddefoot JR, Hinson JP, Clark AJL and Vinson GP (1993*a*) A monoclonal antibody to a conserved sequence in the extracellular domain recognises the angiotensin II AT1 receptor in mammalian target tissues. *J Mol Endocrinol* **11**: 241–245

Barker S, Marchant W, Clark AJL, Jimenez E, Marsigliante S, Montiel M and Vinson GP (1993*b*) Comparison of cos cell transfected AT (1A) and AT (1B) angiotensin II receptors and angiotensin II receptor isoforms in rat tissues using isoelectric focusing. *Biochem Biophys Res Commun* **192**: 392–398

Capponi AM and Catt KJ (1980) Solubilization and characterization of adrenal and uterine angiotensin II receptors after photoaffinity labeling. *J Biol Chem* **255**: 12081–12086

Chen LI, Prakash OM and Re RN (1993) The interaction of insulin and angiotensin II on the regulation of human neuroblastoma cell growth. *Mol Chem Neuropathol* **18**: 189–196

Desarnaud F, Marie J, Lombard C, Larguier R, Seyer R, Lorca T, Jard S and Bonnafous J-C (1993) Deglycosylation and fragmentation of purified rat liver angiotensin II receptor: application to the mapping of hormone-binding domains. *Biochem J* **289**: 289–297

Dzau VJ (1993) Tissue renin–angiotensin system in myocardial hypertrophy and failure. *Arch Int Med* **153**: 937–942

Herblin WF, Chiu AT, McCall DE, Ardecky RJ, Carini DJ, Duncia JV, Pease LJ, Wong PC, Wexler RR, Johnson AL and Timmermans PBMWM (1991) Angiotensin II receptor heterogeneity. *Am J Hypertens* **4**: 299S–302S

Laemmli UK (1970) Cleavage of structural proteins during the assembly of the head of bacteriophage T4. *Nature* **227**: 680–685

Lowry OH, Rosenbrough NJ, Farr AL and Randall RJ (1951) Protein measurement with the folin phenol reagent. *J Biol Chem* **192**: 265–275

Mendelson FAD, Quirion R, Saavedra JM, Aguilera G and Catt KJ (1984) Autoradiographic localization of angiotensin II receptors in rat brain. *Proc Natl Acad Sci USA* **81**: 1575–1579

Noguchi S, Miyauci K, Nishizawa Y, Sasaki Y, Imaoka S, Iwanaga T, Koyama H and Terasawa T (1988) Augmentation of anticancer effect with angiotensin II in intraarterial infusion chemotherapy of breast carcinoma. *Cancer* **62**: 467–473

Okura T, Kitami Y, Wakamiya R, Marumoto K, Iwata T and Hiwada K (1992) Renal and extra renal renin gene expression in spontaneously hypertensive rats. Blood Pressure **3**(suppl.): 6–11

Ouali R, Poulette S, Penhoat A and Saez JM (1992) Characterization and coupling of angiotensin II receptor subtypes in cultured bovine adrenal fasciculata cells. *J Steroid Biochem Mol Biol* **43**: 271–280

Peach MT (1977) Renin–angiotensin system: biochemistry and mechanism of action. *Physiol Rev* **57**: 313–370

Saridogan E, Djahanbakhch O, Puddefoot JR, Demetroulis C, Collingwood K, Mehta JG and Vinson GP (1996*a*) Angiotensin II receptors and angiotensin II stimulation of ciliary activity in human fallopian tube. *J Clin Endocrinol Metab* **81**: 2719–2725

Saridogan E, Djahanbakhch O, Puddefoot JR, Demetroulis C, Dawda R, Hall AJ and Vinson GP (1996*b*) Type 1 angiotensin II receptors in human endometrium. *Mol Hum Reprod* **2**: 659–664

Scatchard G (1949) The attractions of proteins for small molecules and ions. *Ann N Y Acad Sci* **51**: 660–670

Sitzman JV, Wu Y and Cameron JL (1994) Altered angiotensin receptors in human hepatocellular and hepatic metastatic colon cancers. *Ann Surg* **219**: 500–507

© Cancer Research Campaign 1997

Trolliet MR and Phillips MI (1992) The effect of chronic bilateral nephrectomy on plasma and brain angiotensin. *J Hypertens* **10**: 29–36

Ullian ME and Linas SL (1989) Role of receptor cycling in the regulation of angiotensin II surface receptor number and angiotensin II uptake in rat vascular smooth muscle cells. *J Clin Invest* **8**: 840–846

Varela AS and Saez JJ (1993) Utility of serum activity of angiotensin converting enzyme as a tumour marker. *Oncology* **50**: 430–435

Vinson GP, Whitehouse BJ and Hinson JP (1992) *The Adrenal Cortex.* Prentice Hall: Englefield Heights.

Vinson GP, Ho MM and Puddefoot JR (1995*a*) The distribution of angiotensin II type I receptors, and the tissue renin–angiotensin systems. *Mol Med Today* **1**: 35–38

Vinson GP, Puddefoot JR, Ho MM *et al* (1995*b*) Type 1 angiotensin II receptors in rat and human sperm. *J Endocrinol* **144**: 1–10

Wang Y, Yamaguchi T, Franco-Saenz R and Mulrow PJ (1992) Regulation of renin gene expression in rat adrenal zona glomerulosa cells. *Hypertension* **20**: 776–781

Whitebread S, Mele M, Kamber B and De Gasparo M (1989) Preliminary biochemical characterization of two angiotensin II receptor subtypes. *Biochem Biophys Res Commun* **163**: 284–291

© Cancer Research Campaign 1997