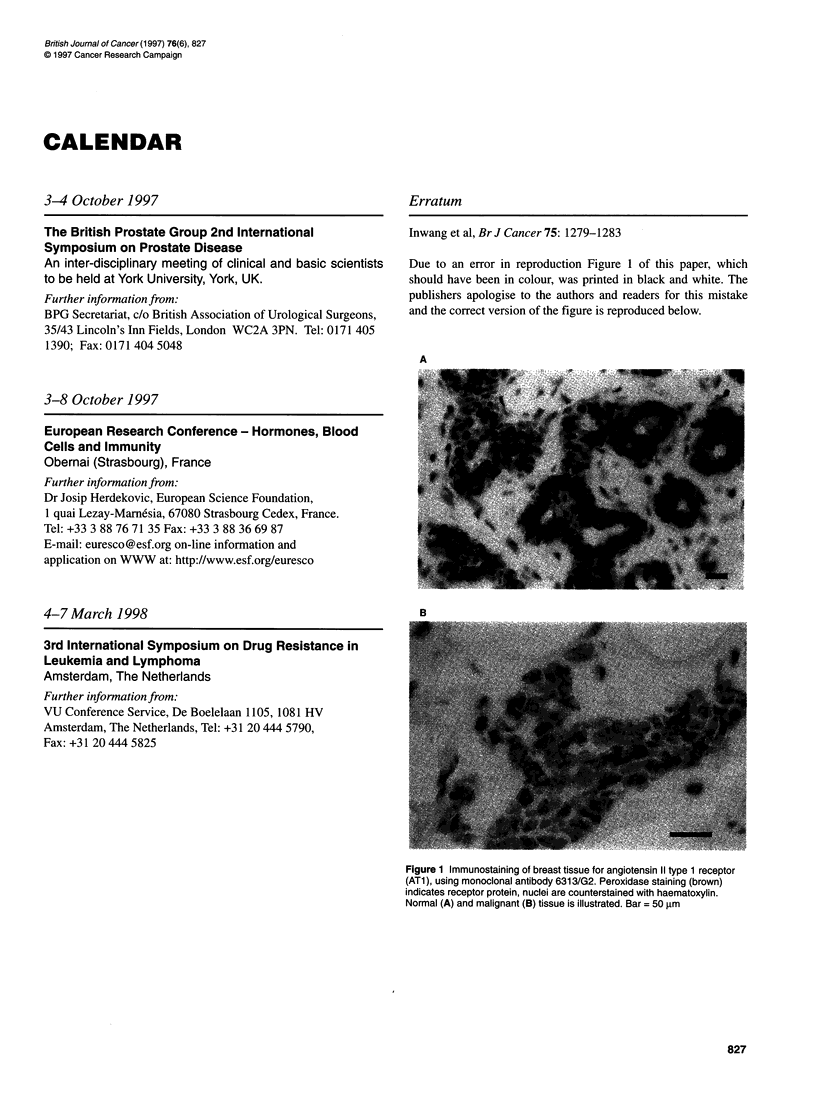# Erratum

**Published:** 1997

**Authors:** 

## Abstract

**Images:**


### Erratum

Inwang et al, *Br J Cancer* **75**: 1279–1283

Due to an error in reproduction Figure 1 of this paper, which should have been in colour, was printed in black and white. The publishers apologise to the authors and readers for this mistake and the correct version of the figure is reproduced below.

**Figure 1** Immunostaining of breast tissue for angiotensin II type 1 receptor (AT1), using monoclonal antibody 6313/G2. Peroxidase staining (brown) indicates receptor protein, nuclei are counterstained with haematoxylin. Normal (**A**) and malignant (**B**) tissue is illustrated. Bar = 50 μm